# Staging discordance in apparent early-stage ovarian neoplasms: prevalence, prognosis, and practical risk stratification

**DOI:** 10.25122/jml-2025-0131

**Published:** 2025-09

**Authors:** Mohamed Abdelwanis Mohamed Abdelaziz, Ambreen Yaseen, Tasrina Akter, Siddesh Prabhulingam, Nesma Hesham, Moustafa Fayad, Hossam Ali

**Affiliations:** 1 Department of Gynecological Oncology, Nottingham University Hospitals NHS Trust, City Campus, Nottingham, United Kingdom; 2 Department of Gynecology and Obstetrics, Nottingham University Hospitals NHS Trust, City Campus, Nottingham, United Kingdom; 3 Department of Gynecology and Obstetrics, Nottingham University Hospitals NHS Trust, Queen's Medical Centre, Nottingham, United Kingdom

**Keywords:** ovarian neoplasm, staging discordance, surgical staging, apparent early-stage disease, risk stratification, clinical decision-making, ascites, CA-125

## Abstract

Up to 1 in 13 patients with apparent early-stage ovarian neoplasms harbor occult advanced disease, posing a diagnostic dilemma with major therapeutic implications that remains poorly characterized. We conducted a retrospective consecutive cohort study of 106 patients with apparent early-stage ovarian neoplasms at a tertiary gynecological oncology center (2014–2023) to determine the prevalence, consequences, and clinical correlates of staging discordance and develop the first descriptive risk stratification for surgical planning. Staging discordance occurred in 8/106 patients (7.5%), all of whom were upstaged to Stage III disease. All malignant cases (5/106, 4.7%) were discordant, demonstrating universally advanced disease requiring chemotherapy (100% vs. 1.0% concordant, *P* < 0.001). A five-year follow-up revealed nearly a five-fold higher recurrence rate, indicating a worse prognosis in discordant cases (37.5% vs 8.2%, *P* = 0.025). Two preoperative features—CA-125 ≥100 U/mL and ascites—were most strongly associated with discordance (both *P* < 0.01). Risk grouping by these factors showed clear stratification: 1.5% discordance with neither factor, 9.5% with one, and 83.3% with both. In this comprehensive consecutive cohort of apparent early-stage ovarian neoplasms, staging discordance was rare but clinically decisive, identifying patients with universally advanced disease, chemotherapy requirement, and worse prognosis. Two readily available preoperative features offer immediate, pragmatic risk stratification to guide surgical triage, particularly in community or resource-limited settings.

## Introduction

Ovarian neoplasms presenting as apparent early-stage disease—lesions appearing confined to the ovary on preoperative assessment without confirmed histological diagnosis—represent one of the most challenging scenarios in gynecological oncology [1,2]. The clinical dilemma centers on determining which patients harbor occult advanced disease despite preoperative appearances suggesting localized pathology, as this fundamentally alters surgical approach, treatment planning, and subspecialty referral patterns [3].

Staging discordance, defined as the discovery of extraovarian disease during surgery in patients with apparent early-stage presentation, represents a critical failure of preoperative assessment with significant clinical consequences [4]. Such discordance may result in inadequate initial surgical preparation, suboptimal procedures by general gynecologists, and potential compromise of patient outcomes [5]. Conversely, accurate risk stratification allows for appropriate surgical planning, informed patient consent, and optimal resource allocation [6].

The biological basis for staging discordance lies in the unique characteristics of ovarian neoplasm dissemination patterns. The dualistic model of ovarian carcinogenesis describes Type I tumors (low-grade serous carcinomas and borderline tumors) that demonstrate early propensity for microscopic peritoneal dissemination through implants undetectable by conventional imaging [7,8]. Borderline ovarian tumors represent a particular challenge, as they may appear entirely benign on imaging yet develop invasive implants that fundamentally alter prognosis and treatment requirements [9,10].

Current preoperative evaluation combines clinical assessment, cross-sectional imaging, and serum tumor markers, primarily CA-125 [11]. Despite technological advances, these modalities demonstrate variable accuracy in detecting occult extraovarian disease, particularly in the absence of obvious metastatic spread [12]. The challenge is further complicated by the heterogeneous nature of ovarian pathology, where borderline tumors may present with invasive implants despite apparent early-stage primary lesions [13].

The clinical implications extend beyond academic interest. Recent evidence demonstrates superior outcomes when ovarian cancer patients are managed initially by gynecological oncologists compared to those requiring secondary referral [14,15]. However, the challenge lies in identifying which apparent early-stage cases harbor occult advanced disease requiring subspecialty expertise from the outset [16]. Recent studies by Matsuo *et al*. reported higher intraoperative capsule rupture rates during minimally invasive surgery in a very large retrospective analysis of 8,850 early ovarian cancer patients, which were associated with worse oncological outcomes, emphasizing the importance of careful patient selection and intraoperative procedures to minimize the risk of tumor disruption and spillage [17]. Similarly, Gallotta *et al.*, in a large international population, suggest that minimally invasive surgery can be offered in appropriately selected early-stage ovarian cancer patients, since pathological and molecular features may be more important than surgical approach to impact survival [18].

Previous studies examining staging accuracy have predominantly focused on confirmed ovarian cancers or specific histological subtypes, with limited attention to the broader spectrum of apparent early-stage ovarian neoplasms [19,20]. Most published research focuses on post-diagnosis staging refinement rather than addressing the fundamental challenge of preoperative risk assessment in cases with uncertain diagnosis [21,22]. Unlike prior studies limited to confirmed ovarian cancer, our consecutive cohort specifically addresses the broader, diagnostically uncertain group of apparent early-stage ovarian neoplasms, where risk stratification is most clinically needed but least studied.

Unlike exploratory prediction models that require external validation, our study provides real-world, consecutive cohort evidence on the frequency, consequences, and recognizable clinical features of staging discordance. To our knowledge, this is the first consecutive cohort study to systematically quantify staging discordance across all apparent early-stage neoplasms (benign, borderline, malignant), directly addressing the clinical blind spot where universal diagnostic uncertainty creates the greatest need for practical guidance.

The primary objective of this study was to quantify the prevalence of staging discordance in apparent early-stage ovarian neoplasms and identify readily available clinical factors that could stratify risk for surgical planning and subspecialty referral decisions. Secondary objectives included describing the clinical consequences and long-term outcomes of staging discordance.

## Material and Methods

### Study design and setting

This single-center retrospective consecutive cohort study analyzed all patients with apparent early-stage ovarian neoplasms treated at Nottingham University Hospitals NHS Trust between January 2014 and December 2023. The study was registered as a service evaluation project and approved by the Clinical Audit and Service Evaluation Department (registration number 25-342C). According to UK Health Research Authority guidelines, formal ethical approval was not required for this retrospective analysis, which utilized anonymized data.

### Study population

**Inclusion criteria:** Adult patients aged ≥18 years; suspected ovarian neoplasm with apparent early-stage disease on preoperative assessment; disease appearing confined to the ovary/ovaries on preoperative imaging and clinical evaluation; no confirmed histological diagnosis preoperatively; primary surgery performed at our institution with comprehensive staging; complete preoperative and surgical staging data available; minimum 6 months follow-up data.

**Exclusion criteria:** Prior history of gynecological malignancy; concurrent gynecological malignancy at diagnosis; obvious advanced-stage disease on preoperative imaging; confirmed tissue diagnosis prior to definitive surgery; incomplete surgical staging or medical records; surgery performed at external institutions.

### Definitions

1. **Apparent early-stage disease:** Ovarian neoplasms meeting ALL criteria: (1) Preoperative imaging (CT/MRI) showing disease confined to ovary/ovaries as assessed through weekly gynecological oncology multidisciplinary team (MDT) meetings including subspecialist radiologists and gynecological oncologists, (2) No obvious extraovarian spread on imaging, (3) No clinical evidence of advanced disease, (4) No confirmed tissue diagnosis preoperatively, and (5) Planned for primary surgical management.
2. **Staging discordance:** Discovery of extraovarian disease spread (Stage II, III, or IV) during surgical staging in patients with apparent early-stage presentation.
3. **Staging concordance:** Final surgical-pathological staging confirming early-stage disease (Stage I) or benign pathology in patients with apparent early-stage presentation.
4. **Serous-type histology:** Pure serous carcinoma, borderline serous tumors, or mixed carcinomas containing serous components.

### Data collection

Comprehensive data extraction included patient demographics, clinical presentation, preoperative imaging findings, serum CA-125 levels, surgical approach and staging details, intraoperative findings, final histopathology, FIGO staging, adjuvant treatment decisions, and follow-up outcomes. The CA-125 threshold of ≥100 U/mL was selected based on both literature evidence and analysis of our cohort data. Studies have shown that women's risk of ovarian cancer diagnosis was increased 205-fold if serum CA-125 was >100 U/mL compared to 36-fold for >30 U/mL, with higher specificity (96.6%) for elevated thresholds. Population-based studies have demonstrated that substantially higher CA-125 levels (89-104 U/mL) are required to reach clinically meaningful cancer probability thresholds, particularly supporting the use of higher cutoffs for staging risk assessment rather than general screening [23,24]. Our preliminary ROC analysis confirmed that a threshold of 100 U/mL provided optimal discrimination for staging discordance (AUC = 0.79), consistent with published evidence that higher cutoffs (89–104 U/mL) improve specificity for staging risk assessment compared to lower thresholds (Supplementary Figure 1).

### Statistical analysis

Variables with clinical relevance were assessed for associations with staging discordance using exact statistical methods. Categorical variables were compared using Fisher's exact test. Continuous variables were analyzed using the Mann-Whitney U test for medians and the *t*-test for means. Given the small number of events, analyses were limited to descriptive associations to avoid overfitting.

Missing data for CA-125 (13.2% of patients) were handled using complete case analysis for the primary risk stratification analysis. Sensitivity analysis was performed to assess the impact of missing data on study conclusions by comparing baseline characteristics between patients with and without available CA-125 data.

Based on univariable associations, we developed a descriptive clinical grouping combining the two factors most strongly associated with discordance. This grouping is presented as a descriptive stratification of observed clinical patterns, rather than as a formal predictive model that requires external validation.

Statistical significance was set at *P* < 0.05. All analyses were performed using SPSS version 28.0 and R version 4.3.0.

## Results

### Patient characteristics

A total of 106 consecutive patients with apparent early-stage ovarian neoplasms met the inclusion criteria. The mean age was 52.5 years (range 15–86 years), with 29 patients (27.3%) aged ≥60 years. All patients presented with disease appearing confined to the ovary on preoperative assessment, with a complete absence of preoperative histological confirmation, necessitating surgical staging based solely on clinical and imaging assessments (Table 1). Comprehensive surgical staging was performed in 90 patients (84.9%), including systematic peritoneal assessment, omentectomy, and lymph node evaluation as clinically indicated.

**Table 1:** Patient demographics and clinical characteristics

Characteristic	All patients (*n* = 106)
**Age (years)**	
**Mean ± SD**	52.5 ± 14.3
**Range**	15-86
**<50 years, *n* (%)**	45 (42.5%)
**50–60 years, *n* (%)**	32 (30.2%)
**≥60 years, *n* (%)**	29 (27.3%)
**CA-125 (U/mL)**	
**Available data, *n* (%)**	92 (86.8%)
**Median (range)**	53 (5-24,862)
**<35, *n* (%)**	39 (42.4%)
**35–100, *n* (%)**	28 (30.4%)
**≥100, *n* (%)**	25 (27.2%)
**Disease Laterality**	
**Unilateral, *n* (%)**	89 (84.0%)
**Bilateral, *n* (%)**	17 (16.0%)
**Ascites Present**	
**Yes, *n* (%)**	18 (17.0%)
**No, *n* (%)**	88 (83.0%)

### Staging discordance analysis

Among 106 patients with apparent early-stage ovarian neoplasms, staging discordance occurred in eight patients (7.5%), all of whom were upstaged to Stage III disease. The remaining 98 patients (92.5%) had staging concordance: 92 patients had Stage I borderline tumors, and six patients had benign lesions.

Malignant neoplasms were identified in five patients (4.7% prevalence). Critically, all malignant cases demonstrated staging discordance to Stage III disease, representing 100% concordance between malignancy and advanced staging in apparent early-stage presentations.

### Clinical impact of staging discordance

The therapeutic implications of staging discordance were profound:

- **Adjuvant chemotherapy:** All discordant cases required chemotherapy, consistent with their upstaging to advanced disease, compared to 1/98 concordant cases (100% vs 1.0%, *P* < 0.001)
- **Recurrence rates:** 37.5% (3/8) in discordant versus 8.2% (8/98) in concordant cases, *P* = 0.025
- **Median follow-up:** 58 months (range 12–118 months)

### Characteristics of staging discordance cases

Detailed analysis of the eight staging discordance cases revealed consistent patterns (Table 2).

**Table 2:** Histological distribution and clinical features of discordant cases

Case	Age	CA-125 (U/mL)	Ascites	Final Histology	Final Stage	Discordance Mechanism
1	75	3,583	Yes	Borderline serous	IIIC	Invasive implants
2	18	15	No	Borderline serous	IIIA	Non-invasive implants
3	84	73	No	High-grade mixed carcinoma	IIIB	Microscopic peritoneal implants
4	15	Not recorded	No	Low-grade serous carcinoma	IIIC	Microscopic lymph node metastases
5	18	60	No	Low-grade serous carcinoma	IIIC	Extensive microscopic peritoneal disease
6	40	<5	No	Borderline serous	IIIB	Invasive omental implants
7	52	552	Yes	Low-grade serous carcinoma	IIIB	Omental metastases
8	55	722	Yes	Low-grade serous carcinoma	IIIA	Microscopic peritoneal disease

**Histological distribution:** Low-grade serous carcinoma: 4/8 (50.0%); Borderline serous tumors with invasive implants: 3/8 (37.5%); High-grade mixed carcinoma: 1/8 (12.5%).

**Mechanisms of discordance:** Microscopic peritoneal implants not detected on imaging: 5/8 (62.5%); Invasive implants in borderline tumours: 3/8 (37.5%); Lymph node metastases: 2/8 (25.0%).

**Clinical factor associations**

Two clinical factors showed the strongest and most consistent associations with staging discordance: a CA-125 level of≥100 U/mL and the presence of ascites. These factors demonstrated both statistical significance and clinically meaningful effect sizes (Table 3).

**Table 3:** Clinical factors associated with staging discordance

Variable	Concordance (*n* = 98)	Discordance (*n* = 8)	*P* value
Age ≥60 years, *n* (%)	24 (24.5%)	5 (62.5%)	0.024*
Mean age ± SD (years)	51.8 ± 14.2	59.4 ± 12.1	0.067†
CA-125 ≥100 U/mL, *n* (%)	19 (21.6%)	6 (75.0%)	0.003*
Median CA-125 (U/mL)	45	387	0.021‡
Bilateral disease, *n* (%)	14 (14.3%)	3 (37.5%)	0.072*
Ascites present, *n* (%)	12 (12.2%)	6 (75.0%)	<0.001*
Median tumour size (cm)	8.2	9.7	0.234‡
Serous-type histology, *n* (%)	66 (67.3%)	8 (100%)	0.030*

* Fisher's exact test; †t-test; ‡Mann-Whitney U test

### Observed risk patterns and clinical grouping

We illustrated the observed stratification of risk by two clinical features. Discordance rates increased stepwise across risk groups (*n* = 92 with complete data):

- **Low risk (0 factors present):** 65 patients, discordance rate 1.5% (1/65)
- **Intermediate risk (1 factor present):** 21 patients, discordance rate 9.5% (2/21)
- **High risk (2 factors present):** 6 patients, discordance rate 83.3% (5/6)

The marked separation demonstrates the clinical utility of these simple features for identifying high-risk patients (Table 4).

**Table 4:** Pragmatic risk grouping for clinical triage

Risk Category	Clinical Features	Discordance Rate	Recommended Management
Low Risk	Neither CA-125 ≥100 nor ascites	1.5% (1/65)	Standard staging with comprehensive peritoneal assessment; General gynecology management acceptable with adequate staging expertise
Intermediate Risk	Either CA-125 ≥100 OR ascites	9.5% (2/21)	Enhanced staging with systematic peritoneal biopsies; Consider subspecialty consultation for surgical planning
High Risk	Both CA-125 ≥100 AND ascites	83.3% (5/6)	Comprehensive staging mandatory; Gynecological oncology management essential; Preoperative counseling for high likelihood of advanced disease

This pragmatic triage pathway offers a straightforward decision-making tool for general gynecologists regarding subspecialty referrals, with particular utility in community or resource-limited settings where complex diagnostic indices may not be readily available.

Two-factor grouping demonstrated improved sensitivity (87.5% vs. 75.0%) and maintained specificity (78.3% vs. 78.6%) compared to CA-125 alone (Supplementary Figure 1). The addition of ascites to CA-125 improved discriminatory ability and clinical utility, demonstrating the value of this simple two-factor approach for identifying high-risk patients.

### Sensitivity analysis results

Analysis of patients with (*n* = 92) versus without (*n* = 14) available CA-125 data revealed no significant differences in baseline characteristics. Mean age was 52.3 years in patients with CA-125 data versus 53.1 years in those without (*P* = 0.85). Histological distribution was similar between groups, with borderline tumors comprising 85.9% of patients with CA-125 data and 92.9% of those without (*P* = 0.71). Staging discordance rates were comparable: 8.7% in patients with CA-125 data versus 0% in those without, although this difference was not statistically significant due to the small sample size (*P* = 0.59). These findings confirm that missing CA-125 data appears to be random and does not introduce systematic bias to our conclusions (Supplementary Table 1).

## Discussion

This consecutive cohort study provides the first comprehensive characterization of staging discordance in apparent early-stage ovarian neoplasms, revealing critical clinical insights with immediate practice implications. Our key findings demonstrate that whilst staging discordance affects 7.5% of apparent early-stage cases, it carries profound therapeutic consequences, with 100% requiring adjuvant chemotherapy and significantly higher recurrence rates.

The high proportion of borderline tumors in our cohort (95/106, 89.6%) reflects the specific clinical scenario we studied – apparent early-stage ovarian neoplasms without preoperative tissue diagnosis. This distribution is characteristic of tertiary referral centers managing diagnostically uncertain ovarian masses, where the majority of truly malignant cases present with obvious advanced disease and are excluded from our "apparent early stage" definition. The high borderline tumor proportion validates our study population as representing the genuine clinical dilemma where staging discordance risk stratification is most needed – cases where preoperative assessment suggests localized disease, but diagnostic uncertainty necessitates comprehensive surgical staging.

### Clinical significance of staging discordance

The 7.5% staging discordance rate, although relatively infrequent, represents patients whose treatment paradigm changed based entirely on surgical findings. This rate falls within the range reported in previous studies of early-stage ovarian cancer, though direct comparison is limited by differences in patient selection and study design [25,26]. The International Collaborative Ovarian Neoplasm (ICON1) and Adjuvant ChemoTherapy in Ovarian Neoplasm (ACTION) trials reported similar upstaging rates in presumed early-stage ovarian cancer [27], whilst institutional series have found variable rates depending on staging protocols [28].

The universal requirement for adjuvant chemotherapy in discordant cases (100% vs 1.0% in concordant cases) validates the critical importance of accurate preoperative risk assessment. The 4.7% malignancy prevalence in apparent early-stage disease, with 100% demonstrating staging discordance, emphasizes that occult advanced disease is the rule rather than the exception when malignancy is present.

Our 5-year follow-up demonstrates that staging discordance is not only a surgical planning issue but also a prognostic marker, with recurrence rates nearly 5-fold higher in discordant cases. This finding supports the clinical relevance of our risk stratification approach and validates the therapeutic decision-making based on comprehensive staging [29,30]. The high rate of comprehensive staging in our cohort (84.9%) reflects appropriate clinical practice given the diagnostic uncertainty inherent in apparent early-stage disease, with systematic approaches to lymphadenectomy being well-established for optimal staging [31].

### Biological basis of staging discordance

The predominance of serous-type histology amongst discordant cases (100% vs 67.3% in concordant cases) aligns with the known biological behavior of ovarian neoplasms [32]. Low-grade serous carcinomas, which comprise 50% of discordant cases, exhibit early microscopic peritoneal dissemination patterns consistent with the dualistic model of ovarian carcinogenesis [33]. The identification of borderline serous tumors with invasive implants in 37.5% of discordant cases highlights the challenge of detecting aggressive biological behavior in apparent early-stage presentations [34,35].

Understanding the patterns of peritoneal dissemination is crucial for comprehending the mechanisms of staging discordance. The peritoneal environment provides an ideal milieu for tumor cell implantation, with gravitational flow patterns directing cells to dependent portions of the peritoneal cavity, including the pouch of Douglas, paracolic gutters, and diaphragmatic surfaces [36]. These microscopic deposits may be present despite normal-appearing imaging, necessitating systematic surgical exploration to exclude occult advanced disease.

The mechanism of implant formation involves exfoliation of tumor cells into the peritoneal cavity, where they can implant on peritoneal surfaces and develop invasive characteristics independent of the primary tumor [37]. This biological behavior explains why comprehensive staging with systematic peritoneal assessment is essential even in apparent early-stage disease.

### Clinical risk patterns

Our consecutive cohort highlights two clinically accessible factors that consistently separated risk groups. This grouping is presented as a descriptive stratification of observed clinical patterns, rather than as a formal predictive model that requires external validation. The marked risk separation achieved—from 1.5% discordance rate in low-risk patients to 83.3% in high-risk patients—offers clear decision-making support for surgical planning and subspecialty referral.

The association with elevated CA-125 is consistent with established literature demonstrating this biomarker's correlation with disease extent [38]. However, our study uniquely applies it to the specific challenge of staging discordance, rather than general malignancy risk. The association with ascites likely reflects microscopic peritoneal disease not detectable by current imaging modalities [39]. This finding aligns with studies demonstrating that even minimal ascites in ovarian neoplasms may indicate more extensive disease than apparent on preoperative assessment.

### Comparison with existing assessment tools

While established tools such as the Risk of Malignancy Index (RMI) and Risk of Ovarian Malignancy Algorithm (ROMA) are designed to distinguish benign from malignant adnexal masses, our approach addresses a fundamentally different clinical question: the risk of staging discordance in apparent early-stage disease, where malignancy status remains unknown [40,41]. RMI, ROMA, and the International Ovarian Tumor Analysis (IOTA) ADNEX model are validated tools for the initial diagnostic question of distinguishing between benign and malignant adnexal masses; however, they serve a fundamentally different purpose than our descriptive risk grouping, which addresses staging uncertainty in apparent early-stage disease. Our approach complements, rather than competes with, these established indices by filling a distinct clinical gap.

The RMI incorporates CA-125, menopausal status, and ultrasound findings to predict malignancy risk, achieving moderate discriminatory ability in distinguishing between benign and malignant masses [42]. ROMA scores combine CA-125 and HE4 with menopausal status to improve malignancy prediction [43]. These tools excel at their intended purpose—identifying which adnexal masses require surgery—but do not address the subsequent challenge of staging risk assessment.

Our findings suggest that two simple, universally available clinical features—CA-125 and ascites—offer striking risk separation for the specific question of staging discordance, potentially more practical in routine or resource-limited settings than complex indices requiring HE4, advanced imaging, or molecular profiling.

### Study strengths and clinical relevance

This represents a comprehensive consecutive institutional series addressing staging discordance specifically in apparent early-stage ovarian neoplasms. Key strengths include:

- Consecutive cohort design minimizing selection bias and providing representative institutional experience
- Comprehensive staging in 84.9% of patients, ensuring complete disease assessment
- Multidisciplinary team assessment, ensuring standardized imaging interpretation by subspecialist radiologists and gynecological oncologists
- Long-term follow-up (median 58 months), validating clinical outcomes and recurrence patterns
- Complete absence of preoperative histological diagnosis represents real-world clinical scenarios where diagnostic uncertainty is universal

The single-center design, whilst potentially limiting generalizability, provides methodological consistency in imaging interpretation, surgical techniques, and pathological assessment. Our tertiary referral center setting may enrich the cohort with more complex cases, but this bias paradoxically strengthens clinical relevance, as complex apparent early-stage cases are precisely those requiring subspecialty expertise and risk stratification.

### Study limitations and future directions

Several limitations warrant acknowledgement and inform future research priorities:

#### Statistical and methodological limitations

- Small number of discordant cases (*n* = 8) reflects the genuine clinical frequency of this phenomenon rather than a methodological shortcoming. Our aim was not to construct or validate a prediction model but to describe the frequency, consequences, and clinical correlates of staging discordance in a real-world consecutive cohort. This descriptive grouping avoids the overfitting risk inherent in small datasets whilst providing immediate clinical utility.
- Single-center retrospective design may limit generalizability to diverse practice settings and healthcare systems.
- The ten-year study period encompasses technological evolution in imaging and surgical techniques, although the consistent application of clinical factors supports robustness.

#### Clinical and practical limitations

- Tertiary referral center bias may not reflect community practice patterns and case complexity.
- Missing CA-125 data in 13.2% of patients, though sensitivity analysis confirmed result stability.
- Retrospective ascites assessment, based on imaging and surgical records, may underestimate the true prevalence.

#### Generalizability considerations

- Limited demographic diversity and predominance of borderline tumors may restrict applicability to other populations.
- A single healthcare system experience may not accurately represent international practice variations.
- Resource availability for comprehensive staging varies across different healthcare settings.

### Future research priorities

Our findings establish several critical research directions with clear pathways for clinical translation:

#### External validation requirements

1. Multi-center prospective studies in diverse patient populations across different healthcare systems and geographic regions
2. Larger sample sizes to improve the precision of association estimates and enable robust multivariable analysis
3. Standardized imaging protocols to reduce inter-observer variability and improve reproducibility across centers
4. Integration with molecular markers such as HE4, tissue-based biomarkers, or genetic profiling to enhance clinical applicability

Future studies in larger multi-center cohorts should assess generalizability; however, our findings should be validated. Recognizing their immediate applicability makes them valuable now for clinical decision-making in this challenging population.

## Conclusion

Staging discordance affects 7.5% of apparent early-stage ovarian neoplasms, representing a rare but clinically decisive event with profound therapeutic implications, including universal requirement for adjuvant chemotherapy and significantly higher recurrence rates. The 4.7% malignancy prevalence in apparent early-stage disease emphasizes the critical importance of comprehensive staging and subspecialty expertise.

Our study identifies two readily available clinical features (CA-125 and ascites) that provide striking stepwise risk separation across all apparent early-stage neoplasms, offering immediately applicable guidance for surgical planning and referral. This represents the first demonstration of such marked risk stratification (1.5% to 83.3% discordance rates) using universally available markers, with particular utility in community or resource-limited settings.

This approach addresses a critical gap in current clinical practice by providing evidence-based guidance for risk-adapted management of apparent early-stage ovarian neoplasms. Our findings establish the first pragmatic triage pathway for this diagnostically challenging population, linking preoperative risk stratification with long-term prognostic outcomes.

While our findings require validation in diverse clinical settings, they establish the foundation for evidence-based risk stratification in this challenging patient population and provide a clinically applicable approach that warrants prospective evaluation.

## Supplementary Material



## Data Availability

Further data is available from the corresponding author upon reasonable request.

## Personal thanks

The authors thank the staff of the medical records department and the pathology department for their assistance with data collection.

## References

[1] Fischerova D, Zikan M, Dundr P, Cibula D (2012). Diagnosis, treatment, and follow-up of borderline ovarian tumours. Oncologist.

[2] Trillsch F, Mahner S, Ruetzel J, Harter P, Ewald-Riegler N, Jaenicke F (2010). Clinical management of borderline ovarian tumors. Expert Rev Anticancer Ther.

[3] Colombo N, Sessa C, du Bois A, Ledermann J, McCluggage WG McNeish, ESMO-ESGO Ovarian Cancer Consensus Conference Working Group (2019). ESMO-ESGO consensus conference recommendations on ovarian cancer: pathology and molecular biology, early and advanced stages, borderline tumours and recurrent disease†. Ann Oncol.

[4] Zanetta G, Rota S, Chiari S, Bonazzi C, Bratina G, Mangioni C (2001). Behavior of borderline tumors with particular interest to persistence, recurrence, and progression to invasive carcinoma: a prospective study. J Clin Oncol.

[5] Bristow RE, Tomacruz RS, Armstrong DK, Trimble EL, Montz FJ (2002). Survival effect of maximal cytoreductive surgery for advanced ovarian carcinoma during the platinum era: a meta-analysis. J Clin Oncol.

[6] Vergote I, Tropé CG, Amant F, Kristensen GB, Ehlen T, Johnson N, European Organization for Research and Treatment of Cancer-Gynaecological Cancer Group; NCIC Clinical Trials Group (2010). Neoadjuvant chemotherapy or primary surgery in stage IIIC or IV ovarian cancer. N Engl J Med.

[7] Kurman RJ, Shih IeM (2016). The dualistic model of ovarian carcinogenesis. Am J Pathol.

[8] Longacre TA, McKenney JK, Tazelaar HD, Kempson RL, Hendrickson MR (2005). Ovarian serous tumors of low malignant potential (borderline tumors): outcome-based study of 276 patients with long-term (> or =5-year) follow-up. Am J Surg Pathol.

[9] Shih KK, Zhou Q, Huh J, Morgan JC, Iasonos A, Aghajanian C (2011). Risk factors for recurrence of ovarian borderline tumors. Gynecol Oncol.

[10] Seidman JD, Kurman RJ (2000). Ovarian serous borderline tumors: a critical review of the literature with emphasis on prognostic indicators. Hum Pathol.

[11] Jacobs I, Oram D, Fairbanks J, Turner J, Frost C, Grudzinskas JG (1990). A risk of malignancy index incorporating CA 125, ultrasound and menopausal status for the accurate preoperative diagnosis of ovarian cancer. Br J Obstet Gynaecol.

[12] Morice P, Uzan C, Fauvet R, Gouy S, Duvillard P, Darai E (2012). Borderline ovarian tumour: pathological diagnostic dilemma and risk factors for invasive or lethal recurrence. Lancet Oncol.

[13] Tropé CG, Kaern J, Davidson B (2012). Borderline ovarian tumours. Best Pract Res Clin Obstet Gynaecol.

[14] Vernooij F, Heintz P, Witteveen E, van der Graaf Y (2007). The outcomes of ovarian cancer treatment are better when provided by gynecologic oncologists and in specialized hospitals: a systematic review. Gynecol Oncol.

[15] Earle CC, Schrag D, Neville BA, Yabroff KR, Topor M, Fahey A (2006). Effect of surgeon specialty on processes of care and outcomes for ovarian cancer patients. J Natl Cancer Inst.

[16] Chi DS, Franklin CC, Levine DA, Akselrod F, Sabbatini P, Jarnagin WR (2004). Improved optimal cytoreduction rates for stages IIIC and IV epithelial ovarian, fallopian tube, and primary peritoneal cancer: a change in surgical approach. Gynecol Oncol.

[17] Matsuo K, Chang EJ, Matsuzaki S, Mandelbaum RS, Matsushima K, Grubbs BH (2020). Minimally invasive surgery for early-stage ovarian cancer: Association between hospital surgical volume and short-term perioperative outcomes. Gynecol Oncol.

[18] Gallotta V, Jeong SY, Conte C, Trozzi R, Cappuccio S, Moroni R, Ferrandina G, Scambia G, Kim TJ, Fagotti A (2021). Minimally invasive surgical staging for early stage ovarian cancer: A long-term follow up. Eur J Surg Oncol.

[19] Vasconcelos I, de Sousa Mendes M (2015). Conservative surgery in ovarian borderline tumours: a meta-analysis with emphasis on recurrence risk. Eur J Cancer.

[20] Daraï E, Fauvet R, Uzan C, Gouy S, Duvillard P, Morice P (2013). Fertility and borderline ovarian tumor: a systematic review of conservative management, risk of recurrence and alternative options. Hum Reprod Update.

[21] du Bois A, Ewald-Riegler N, de Gregorio N, Reuss A, Mahner S, Fotopoulou C, Arbeitsgmeinschaft Gynäkologische Onkologie (AGO) Study Group (2013). Borderline tumours of the ovary: A cohort study of the Arbeitsgmeinschaft Gynäkologische Onkologie (AGO) Study Group. Eur J Cancer.

[22] Delle Marchette M, Ceppi L, Andreano A, Bonazzi CM, Buda A, Grassi T, Giuliani D, Sina F, Lamanna M, Bianchi T, Lissoni AA, Landoni F, Valsecchi MG, Fruscio R (2019). Oncologic and fertility impact of surgical approach for borderline ovarian tumours treated with fertility sparing surgery. Eur J Cancer.

[23] Jacobs IJ, Menon U, Ryan A, Gentry-Maharaj A, Burnell M, Kalsi JK (2016). Ovarian cancer screening and mortality in the UK Collaborative Trial of Ovarian Cancer Screening (UKCTOCS): a randomised controlled trial. Lancet.

[24] Funston G, Hamilton W, Abel G, Crosbie EJ, Rous B, Walter FM (2020). The diagnostic performance of CA125 for the detection of ovarian and non-ovarian cancer in primary care: A population-based cohort study. PLoS Med.

[25] Lee JY, Kim HS, Chung HH, Kim JW, Park NH, Song YS (2014). The role of omentectomy and random peritoneal biopsies as part of comprehensive surgical staging in apparent early-stage epithelial ovarian cancer. Ann Surg Oncol.

[26] Panici PB, Maggioni A, Hacker N, Landoni F, Ackermann S, Campagnutta E (2005). Systematic aortic and pelvic lymphadenectomy versus resection of bulky nodes only in optimally debulked advanced ovarian cancer: a randomized clinical trial. J Natl Cancer Inst.

[27] Trimbos JB, Parmar M, Vergote I, Guthrie D, Bolis G, Colombo N, European Organisation for Research and Treatment of Cancer Collaborators-Adjuvant ChemoTherapy un Ovarian Neoplasm (2003). International Collaborative Ovarian Neoplasm trial 1 and Adjuvant ChemoTherapy In Ovarian Neoplasm trial: two parallel randomized phase III trials of adjuvant chemotherapy in patients with early-stage ovarian carcinoma. J Natl Cancer Inst.

[28] Young RC, Walton LA, Ellenberg SS, Homesley HD, Wilbanks GD, Decker DG (1990). Adjuvant therapy in stage I and stage II epithelial ovarian cancer. Results of two prospective randomized trials. N Engl J Med.

[29] Walker JL, Piedmonte MR, Spirtos NM, Eisenkop SM, Schlaerth JB, Mannel RS (2009). Laparoscopy compared with laparotomy for comprehensive surgical staging of uterine cancer: Gynecologic Oncology Group Study LAP2. J Clin Oncol.

[30] Trimbos JB, Vergote I, Bolis G, Vermorken JB, Mangioni C, Madronal C, EORTC-ACTION collaborators (2003). European Organisation for Research and Treatment of Cancer-Adjuvant ChemoTherapy in Ovarian Neoplasm. Impact of adjuvant chemotherapy and surgical staging in early-stage ovarian carcinoma: European Organisation for Research and Treatment of Cancer-Adjuvant ChemoTherapy in Ovarian Neoplasm trial. J Natl Cancer Inst.

[31] Maggioni A, Benedetti Panici P, Dell'Anna T, Landoni F, Lissoni A (2006). Randomised study of systematic lymphadenectomy in patients with epithelial ovarian cancer macroscopically confined to the pelvis. Br J Cancer.

[32] Kurman RJ, Shih IeM (2010). The origin and pathogenesis of epithelial ovarian cancer: a proposed unifying theory. Am J Surg Pathol.

[33] Gershenson DM, Sun CC, Bodurka D, Coleman RL, Lu KH, Sood AK, Deavers M, Malpica AL, Kavanagh JJ (2009). Recurrent low-grade serous ovarian carcinoma is relatively chemoresistant. Gynecol Oncol.

[34] Trimble CL, Kosary C, Trimble EL (2002). Long-term survival and patterns of care in women with ovarian tumours of low malignant potential. Gynecol Oncol.

[35] Silva EG, Gershenson DM, Malpica A, Deavers M (2006). The recurrence and the overall survival rates of ovarian serous borderline neoplasms with noninvasive implants is time dependent. Am J Surg Pathol.

[36] Koppe MJ, Boerman OC, Oyen WJ, Bleichrodt RP (2006). Peritoneal carcinomatosis of colorectal origin: incidence and current treatment strategies. Ann Surg.

[37] Lengyel E (2010). Ovarian cancer development and metastasis. Am J Pathol.

[38] Bast RC, Klug TL, St John E, Jenison E, Niloff JM, Lazarus H (1983). A radioimmunoassay using a monoclonal antibody to monitor the course of epithelial ovarian cancer. N Engl J Med.

[39] Ayhan A, Gultekin M, Taskiran C, Dursun P, Firat P, Bozdag G (2007). Ascites and epithelial ovarian cancers: a reappraisal with respect to different aspects. Int J Gynecol Cancer.

[40] Moore RG, McMeekin DS, Brown AK, DiSilvestro P, Miller MC, Allard WJ (2009). A novel multiple marker bioassay utilizing HE4 and CA125 for the prediction of ovarian cancer in patients with a pelvic mass. Gynecol Oncol.

[41] Tingulstad S, Hagen B, Skjeldestad FE, Onsrud M, Kiserud T, Halvorsen T (1996). Evaluation of a risk of malignancy index based on serum CA125, ultrasound findings and menopausal status in the pre-operative diagnosis of pelvic masses. Br J Obstet Gynaecol.

[42] Karlsen MA, Sandhu N, Høgdall C, Christensen IJ, Nedergaard L, Lundvall L (2012). Evaluation of HE4, CA125, risk of ovarian malignancy algorithm (ROMA) and risk of malignancy index (RMI) as diagnostic tools of epithelial ovarian cancer in patients with a pelvic mass. Gynecol Oncol.

[43] Moons KG, Altman DG, Reitsma JB, Ioannidis JP, Macaskill P, Steyerberg EW (2015). Transparent Reporting of a multivariable prediction model for Individual Prognosis or Diagnosis (TRIPOD): explanation and elaboration. Ann Intern Med.

