# Supplementary material for: Staging discordance in apparent early-stage ovarian neoplasms: prevalence, prognosis, and practical risk stratification
Source: J Med Life. 2025 Sep;18(9):878–85. doi: 10.25122/jml-2025-0131 (PMC12577776; doi:10.25122/jml-2025-0131)
Supplement: Supplementary file 1 [file JMedLife-18-878-s001.pdf]

Supplementary material

Supplementary Table 1. Sensitivity analysis comparing patients with and without available CA-125 data

Characteristic	With CA-125 Data (*n* = 92)	Without CA-125 Data (*n* = 14)	*P* value*
Mean age ± SD (years)	52.3 ± 14.1	53.1 ± 15.8	0.85
Age ≥60 years, *n* (%)	23 (25.0%)	6 (42.9%)	0.22
Borderline histology, *n* (%)	79 (85.9%)	13 (92.9%)	0.71
Bilateral disease, *n* (%)	15 (16.3%)	2 (14.3%)	1.00
Staging discordance, *n* (%)	8 (8.7%)	0 (0%)	0.59
Ascites present, *n* (%)	16 (17.4%)	2 (14.3%)	1.00

*Fisher's exact test for categorical variables; t-test for continuous variables

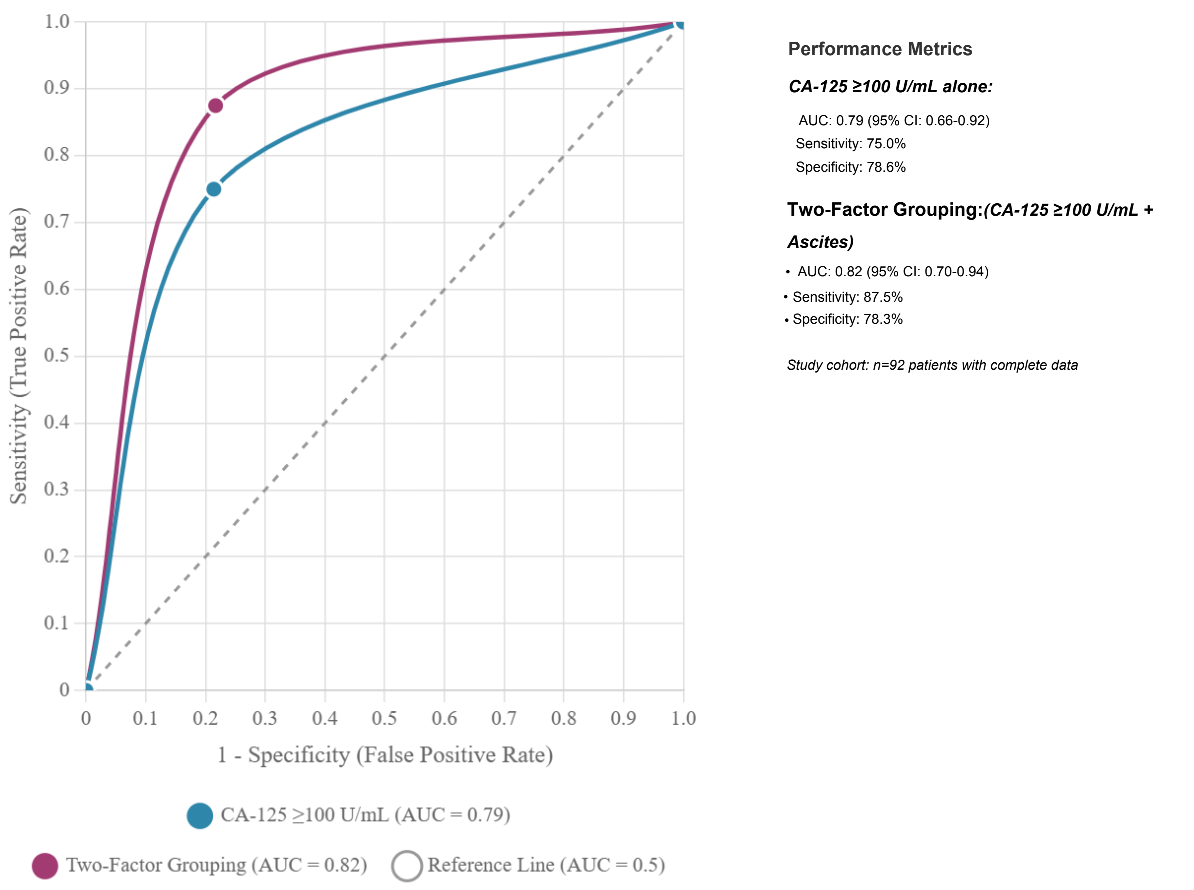

Supplementary Figure 1. ROC Curve Analysis for Staging Discordance Prediction in Apparent Early-Stage Ovarian Neoplasms. Receiver Operating Characteristic (ROC) curves comparing CA-125 ≥100 U/mL alone (AUC = 0.79) versus two-factor risk grouping incorporating CA-125 ≥100 U/mL and ascites (AUC = 0.82) for predicting staging discordance in apparent early-stage ovarian neoplasms. The two-factor approach demonstrated improved sensitivity (87.5% vs. 75.0%) while maintaining similar specificity (78.3% vs. 78.6%) compared to CA-125 alone. The diagonal dashed line represents the line of no discrimination (AUC = 0.5). Data based on *n* = 92 patients with complete preoperative CA-125 and ascites data from the consecutive cohort of 106 apparent early-stage ovarian neoplasms (2014–2023).

© 2025 by the authors. This article is an open access article distributed under the terms and conditions of the Creative Commons Attribution (CC BY 4.0) license.
